# Risk of glucose intolerance and gestational diabetes mellitus in relation to maternal habitual snoring during early pregnancy

**DOI:** 10.1371/journal.pone.0184966

**Published:** 2017-09-19

**Authors:** Chunfang Qiu, Wayne Lawrence, Bizu Gelaye, Lee Stoner, Ihunnaya O. Frederick, Daniel A. Enquobahrie, Tanya K. Sorensen, Michelle A. Williams

**Affiliations:** 1 Center for Perinatal Studies, Swedish Medical Center, Seattle, Washington, United States of America; 2 Department of Epidemiology, Harvard T. Chan School of Public Health, Boston, Massachusetts, United States of America; 3 Department of Sport and Exercise Science, University of North Carolina, Chapel Hill, North Carolina, United States of America; 4 Department of Epidemiology, Cardiovascular Health Research Unit, University of Washington, Seattle, WA, United States of America; Shanghai Jiaotong University School of Medicine Xinhua Hospital, CHINA

## Abstract

**Background:**

Obstructive sleep apnea (OSA) or habitual snoring is known to be associated with impaired glucose tolerance and type 2 diabetes among both men and non-pregnant women. We examined the association of habitual snoring during early pregnancy with risk of impaired glucose tolerance (IGT) and gestational diabetes mellitus (GDM).

**Methods:**

A cohort of 1,579 women was interviewed during early pregnancy. We collected information about snoring frequency during early pregnancy. Results from screening and diagnostic tests for IGT and GDM were abstracted from medical records. Multivariate logistic regression models were fitted to estimate odds ratios (OR) and 95% confidence intervals (95% CI) of IGT and GDM associated with snoring in early pregnancy.

**Results:**

Overall, women who snored “most or all of the time” had a 2.1-fold increased odds of IGT (OR 2.10; 95% CI 1.31–3.35) and a 2.5-fold increased odds of GDM (OR 2.50; 95% CI 1.34–4.67) as compared with women who never snored. Compared with lean women (pre-pregnancy body mass index (BMI) <25 kg/m^2^) who did not snore, lean snorers had a 2-fold increased odds of GDM (OR = 1.99, 95% CI: 1.07–3.68). The odds of GDM risk was particularly elevated among overweight women (BMI ≥ 25 kg/m^2^) who snored (OR = 5.01; 95% CI 2.71–9.26). However, there was no evidence of an interaction between overweight and snoring with GDM risk (p-value = 0.144).

**Conclusions:**

These findings, if confirmed, may have important implications for tailoring prenatal care for overweight pregnant women, and /or those with a history of habitual snoring in early pregnancy.

## Background

A growing body of epidemiologic evidence suggests that sleep-disorder breathing (SDB), characterized by chronic intermittent hypoxia, airflow limitation and recurrent arousals, is an important risk factor for the development of cardiovascular and metabolic complications, including hypertension, stroke, hyperglycemia and type II diabetes [1–6]. Snoring, a common sleep disorder among pregnant women and a symptom of SDB, has been shown to predict the onset of diabetes in non-pregnant women and men [6–7]. However, relatively little is known about the impact of snoring on maternal health during early pregnancy.

While previous studies have independently reported increased incidence of snoring and glucose intolerance during pregnancy [4, 8], a few of studies [4, 9–15] have investigated whether there is an association between snoring during early pregnancy and incident gestational diabetes mellitus (GDM). Some concluded a positive association [4, 11, 13–14] and the others indicated controversial results [9, 10, 12]. For example, Louis et al did not find an association between severe snoring and subsequent GDM in obese pregnant women [10]. If snoring during early pregnancy independently contributes to, or is an indicator of maternal metabolic complications during pregnancy, then improved comprehension of this association may aid in improving both maternal and newborn health. Therefore, the aim of this study was to investigate whether snoring during early pregnancy is associated with clinically diagnosed impaired glucose tolerance and GDM. We further explored the independent and joint effect of snoring and pre-pregnancy overweight status (define as pre-pregnancy body mass index (BMI) ≥ 25 kg/m^2^) on GDM risk.

## Methods

### Study population and procedures

This study was conducted using a cohort of pregnant women enrolled in the Migraine and Pregnancy Study, a prospective cohort study designed to investigate the relationship between migraine and headache symptoms prior to and during pregnancy, and the risk of adverse perinatal outcomes including preeclampsia [16]. Participants were recruited from prenatal care clinics affiliated with Swedish Medical Center in Seattle, WA from November 2009 to March 2013. Women were eligible if they began prenatal care at or prior to 20 weeks gestation, planned to carry the pregnancy to term and deliver at Swedish Medical Center, spoke and read English and were at least 18 years of age. A structured interview was used to obtain information about health, sociodemographic and lifestyle factors. Pregnancy outcome information was abstracted from medical and clinical records by study personnel. All study procedures and protocols were approved by the Institutional Review Board of Swedish Medical Center. All participants provided written consent.

### Analytical population

Of the 2,051 participants that were originally included, 394 (19.2%) were excluded due to incomplete questionnaire data, 69 (3.4%) for answering “Don’t know” to snoring during pregnancy questions, and 9 (0.44%) for unknown GDM case control status. A total of 1,579 (76.9%) participants (all without a history of pre-gestational diabetes) were included in the present analysis. Excluded participants did not differ from the rest of the cohort with respect to sociodemographic or lifestyle characteristics. Excluded participants also had similar GDM incidence (6.4%) as compared with the participants retained for the present analysis (6.3%).

### Description of covariates

Participants completed a questionnaire administered by trained interviewers at enrollment in early pregnancy (15.1 weeks on average, with standard deviation (SD) of 2.6 weeks). Information on maternal sociodemographic characteristics and medical history were obtained using structured interviews. The average maternal self-reported nightly snoring frequency was ascertained by asking women the following question: “Since becoming pregnant, when you are asleep, to the best of your knowledge, have you snored?” Participants response selection were as follows: (i) all of the time, (ii) most of the time, (iii) some of the time, (iv) a little of the time, and (v) none of the time. Pre-pregnancy weight and height were based on self-reports obtained during the interview. Weight in kilograms divided by height in meters squared was used to calculate pre-pregnancy body mass index (BMI). Pre-pregnancy overweight status was defined as pre-pregnancy BMI ≥ 25.0 kg/m^2^.

### Impaired glucose tolerance (IGT) and gestational diabetes mellitus (GDM)

All women were screened at 24–28 weeks gestation using a 50 gram 1-hour oral glucose challenge test, based on American Diabetes Association (ADA) recommendations. Participants who failed the screening test (glucose ≥ 140 mg/dl) were defined as impaired glucose tolerance (IGT). Those IGT subjects were followed up after 1–2 weeks with a 100 gram 3-hour oral glucose tolerance test (OGTT) [17]. Women were diagnosed with GDM if two or more of the 100 gram OGTT glucose levels exceeded the ADA criteria: fasting ≥ 95 mg/dl; 1-hour ≥ 180 mg/dl; 2-hour ≥ 155 mg/dl; 3-hour ≥ 140 mg/dl [17].

### Statistical analytical methods

We compared the frequency distribution of socio-demographic, lifestyle, behavioral and medical history characteristics of participants according to maternal snoring frequency using one-way analysis of variance (ANOVA) and Chi-squared test. Multivariate adjusted logistic regression procedures were used to calculate maximum likelihood estimates of odds ratios (ORs) and 95% confidence intervals (CI) of diagnosed IGT and GDM, respectively, in relation to maternal self-reported early pregnancy snoring frequency. We selected potential confounders from a list of variables that were associated with snoring (from prior studies conducted among men and non-pregnant women) and that met criteria for confounding based on a review of the literature (i.e., maternal age, race) and assessment of potential causal relationships based on prior knowledge. We then controlled for potential confounders that changed multivariable ORs by more than 10% relative to the unadjusted ORs. Linear regression models were fitted to compare mean values of glucose concentration post oral glucose challenge (50 g glucose load) according to the frequency of maternal early pregnancy snoring. Lastly, we evaluated the independent and joint effect of maternal pre-pregnancy overweight status and early pregnancy snoring on risk of incident GDM. For these analyses, we classified women according to the joint distribution of pre-pregnancy lean or overweight status (< 25 vs. ≥ 25 kg/m^2^) and any snoring during early pregnancy (no vs. yes) thus resulting in the following categories: lean and non-snorer (the reference group); lean and snorer; overweight and non-snorer; and overweight and snorer. All analyses were performed using Stata 13.0 statistical software (Stata, College Station, TX). All reported *P* values are two tailed and were deemed statistically significant at α = 0.05.

## Results

Characteristics of the study cohort are summarized in **Table 1**. Overall, participants included in this analysis tended to be Caucasian, well-educated, and married. Frequent snorers were more likely to be older, of non-Hispanic White race/ethnicity, current smokers, have family history of diabetes and heavier than women who did not report frequent snoring. Frequent snorers were also more likely to develop IGT and GDM later in pregnancy.

**Table 1:** Characteristics of the study population, April 2009—June 2013.

		Frequency of Snoring during Early Pregnancy				
Characteristics	Cohort (N = 1579)	None of the time (N = 874)	A little of the time (N = 316)	Some of the time (N = 243)	Most or all of the time (N = 146)	p-value
Maternal Age (years)	33.8 ± 4.1	33.4 ± 3.9	34.2 ± 4.4	34.1 ± 4.1	35.0 ± 4.6	<0.001
Maternal Age (years)						
<35	932 (59.0)	560 (64.1)	173 (54.8)	130 (53.5)	69 (47.3)	<0.001
≥ 35	647 (41.0)	314 (35.9)	143 (45.3)	113 (46.5)	77 (52.7)	
Maternal Race/Ethnicity						
Non-Hispanic White	1287 (81.5)	737 (84.3)	240 (76.0)	202 (83.1)	108 (74.0)	0.004
Asian	180 (11.4)	90 (10.3)	46 (14.6)	23 (9.5)	21 (14.4)	
Other	112 (7.1)	45 (5.4)	30 (9.4)	18 (7.4)	17 (11.6)	
Single Marital Status	125 (7.9)	61 (7.0)	21 (6.7)	27 (11.1)	16 (11.0)	0.074
Nulliparous	820 (51.9)	448 (51.3)	171 (54.1)	129 (53.1)	72 (49.3)	0.731
Cigarette Smoker	61 (3.9)	30 (3.4)	6 (1.9)	15 (6.2)	10 (6.9)	0.013
Family History of Diabetes	338 (21.4)	166 (19.0)	92 (29.1)	51 (21.0)	29 (19.9)	0.002
Pre-pregnancy BMI (kg/m^2^)	23.4 ± 4.4	22.9 ± 3.9	23.5 ± 4.1	24.4 ± 5.1	25.7 ± 5.5	<0.001
Pre-pregnancy BMI (kg/m^2^)						
<18.5	57 (3.6)	38 (4.4)	7 (2.2)	10 (4.1)	2 (1.4)	<0.001
18.5–24.9	1116 (70.7)	658 (75.3)	228 (72.2)	154 (63.4)	76 (52.1)	
25.0–29.9	291 (18.4)	134 (15.3)	61 (19.3)	49 (20.2)	47 (32.3)	
≥ 30.0	115 (7.3)	44 (5.0)	20 (6.3)	30 (12.4)	21 (14.4)	
Gestational Age at Delivery (weeks)	38.9 ± 1.9	38.9 ± 1.9	38.9 ± 1.8	38.9 ± 1.7	38.6 ± 2.1	0.400
Gestational Age at Interview (weeks)	15.1 ± 2.6	15.0 ± 2.6	15.3 ± 2.6	15.1 ± 2.6	15.4 ± 2.8	0.098
Infant Birthweight (kg)	3.4 ± 0.9	3.4 ± 0.5	3.4 ± 1.0	3.5 ± 1.9	3.4 ± 0.6	0.114
Post Load Glucose (mg/dl)*	110.3 ± 29.1	107.5 ± 27.3	112.8 ± 32.2	112.2 ± 29.3	118.0 ± 29.9	<0.001
Glucose Intolerance **	217 (13.7)	92 (10.5)	57 (18.0)	38 (15.6)	30 (20.6)	<0.001
Incident GDM	100 (6.3)	38 (4.4)	29 (9.2)	15 (6.2)	18 (12.3)	<0.001

Data in mean ± SD or number (%)

*p-value from ANOVA one way (continuous variable) or Chi-Square test (categorical variable)

* Post oral glucose challenge (50 g glucose load)

** Glucose Intolerance, post oral glucose challenge screening test glucose ≥ 140 mg/dl

As shown in **Table 2**, after adjusting for maternal age and race/ethnicity, smoking during pregnancy and family history of diabetes, women who reported snoring “most or all of the time” during early pregnancy had 2.1-fold increased odds of IGT (OR = 2.10; 95% CI 1.31–3.35) and a 2.5-fold increased odds of GDM (OR = 2.50; 95% CI 1.34–4.67) compared with women who did not snore (the reference group). These positive associations remained, though slightly attenuated, after further adjustment for maternal pre-pregnancy BMI (OR = 1.77; 95% CI 1.10–2.86, for IGT) and (OR = 1.88; 95% CI 0.99–3.59, for GDM), respectively.

**Table 2:** Risk of impaired glucose tolerance after a 50-Gram oral glucose challenge screening test and incident gestational diabetes mellitus according to maternal snoring during early pregnancy.

	Frequency of Snoring during Early Pregnancy				
Outcomes	None of the time (N = 874)	A little of the time (N = 316)	Some of the time (N = 243)	Most or all of the time (N = 146)	P-value for Linear Trend
**Glucose Intolerance**					
**All, n (%)**	**92 (10.5)**	**57 (18.0)**	**38 (15.6)**	**30 (20.6)**	
Unadjusted OR (95%CI)	1.00 (Referent)	1.87 (1.31–2.68)	1.58 (1.05–2.37)	2.20 (1.39–3.47)	<0.001
*Adjusted OR (95%CI)	1.00 (Referent)	1.70 (1.18–2.45)	1.58 (1.04–2.39)	2.10 (1.31–3.35)	0.001
**Adjusted OR (95%CI)	1.00 (Referent)	1.65 (1.14–2.38)	1.42 (0.94–2.17)	1.77 (1.10–2.86)	0.008
**Gestational Diabetes Mellitus**					
n (%)	38 (4.4)	29 (9.2)	15 (6.2)	18 (12.3)	
Unadjusted OR (95%CI)	1.00 (Referent)	2.22 (1.35–3.67)	1.45 (0.78–2.68)	3.09 (1.71–5.59)	0.001
*Adjusted OR (95%CI)	1.00 (Referent)	1.68 (0.99–2.83)	1.31 (0.69–2.46)	2.50 (1.34–4.67)	0.009
**Adjusted OR (95%CI)	1.00 (Referent)	1.59 (0.93–2.71)	1.11 (0.58–2.12)	1.88 (0.99–3.59)	0.102

*Adjusted by maternal age, race (non-Hispanic White vs. other), smoking during pregnancy and family history of diabetes mellitus.

**Adjusted by maternal age, race (non-Hispanic White vs. other), smoking during pregnancy and family history of diabetes mellitus as well as pre-pregnancy BMI.

**Fig 1:**
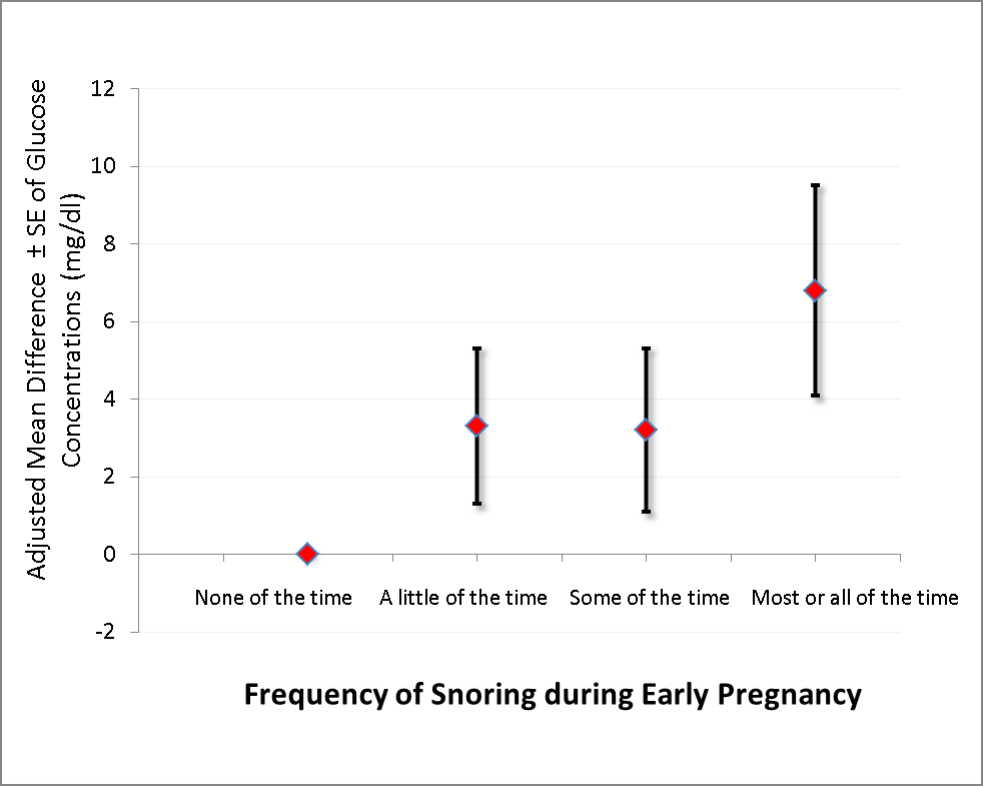
The adjusted mean difference and standard error (SE) bars of glucose concentration according to the frequency of snoring during early pregnancy after adjusting for maternal age, race, smoking during pregnancy and family history of diabetes mellitus as well as pre-pregnancy BMI.

**Table 3** shows results from linear regression model of glucose concentration post oral glucose challenge (50 g glucose load) according to frequency of snoring during early pregnancy. Mean glucose concentrations were 3.7 mg/dl higher in women who reported snoring a little of the time (95% CI -0.1, 7.6), 4.5 mg/dl higher for women who reported snoring some of the time (95% CI 0.5, 8.6), and 9.3 mg/dl higher for women who reported snoring most or all of the time (95% CI 4.2, 14.5) compared with those who reported snoring none of the time (P-value for trend <0.005). After further adjusting for pre-pregnancy BMI the corresponding differences were 3.3 (95% CI -0.5, 7.1), 3.2 (95% CI -0.9, 7.3) and 6.8 (95% CI 1.5, 12.1) across the successive categories of frequency of snoring with women who reported not snoring at all as the reference group (**Fig 1**).

**Table 3:** Linear regression model of glucose concentrations according to frequency of snoring during early pregnancy.

	Frequency of Snoring During Early Pregnancy				
Post Load Glucose (mg/dl)	None of the time	A little of the time	Some of the time	Most or all of the time	P-value for Linear Trend
All	N = 874	N = 316	N = 243	N = 146	
Mean ± SD	107.5 ± 27.3	112.8 ± 32.2	112.2 ± 29.3	118.0 ± 29.9	
Unadjusted β ± SE (95%CI)	Referent	5.3 ± 2.0 (1.3, 9.3)	4.7 ± 2.1 (0.6, 8.8)	10.5 ± 2.6 (5.3, 15.6)	<0.001
*Adjusted β ± SE (95%CI)	Referent	3.7 ± 2.0 (-0.1, 7.6)	4.5 ± 2.1 (0.5, 8.6)	9.3 ± 2.6 (4.2, 14.5)	<0.001
**Adjusted β ± SE (95%CI)	Referent	3.3 ± 2.0 (-0.5, 7.1)	3.2 ± 2.1 (-0.9, 7.3)	6.8 ± 2.7(1.5, 12.1)	0.005

history of diabetes mellitus.

**Adjusted by maternal age, race (non-Hispanic White vs. other), smoking during pregnancy and family history of diabetes mellitus as well as pre-pregnancy BMI.

Finally, we examined the independent and joint associations of pre-pregnancy overweight status and snoring (yes/no) with GDM risk (**Table 4**). Compared with lean women who did not snore, lean women who snored had a 2-fold increased odds of GDM (OR = 1.99, 95% CI: 1.07–3.68). The odds of GDM risk was particularly elevated among overweight women who snored. Compared with lean women who did not snore, those who were overweight and snored had 5-fold (OR = 5.01; 95% CI 2.71–9.26) increased odds of GDM. However, we observed no evidence of a statistically significant interaction of overweight status and snoring with GDM risk (p-value = 0.144).

**Table 4:** Odds ratios (OR) and 95% confidence intervals (95%CI) of gestational diabetes mellitus (GDM) risk according to any snoring during early pregnancy and pre-pregnancy overweight status.

Snore* & pre-pregnancy BMI≥25kg/m^2^	GDM cases (N = 100)	Controls (N = 1479)	Unadjusted OR (95%CI)	Adjusted OR ** (95%CI)
	n (%)	n (%)		
No & No	18 (18.0)	678 (45.8)	1.00 (Referent)	1.00 (Referent)
Yes & No	28 (28.0)	449 (30.4)	2.35 (1.28–4.30)	1.99 (1.07–3.68)
No & Yes	20 (20.0)	158 (10.7)	4.77 (2.46–9.22)	4.83 (2.45–9.51)
Yes & Yes	34 (34.0)	194 (13.1)	6.60 (3.65–11.95)	5.01 (2.71–9.26)
*p-value for interaction term*			*0*.*220*	*0*.*144*

*Definition of snore: any snoring during early pregnancy

**Adjusted by maternal age, race (non-Hispanic White vs. other), smoking during pregnancy and family history of diabetes mellitus.

## Discussion

Findings from this study suggest that women who are frequent snorers in early pregnancy are more likely to later develop IGT and GDM, respectively. Furthermore, compared to lean women who do not snore, lean women who snored had a 2-fold increased odds of GDM; and the odds were particularly elevated for overweight women who snored (OR = 5.01; 95% CI 2.71–9.26).

Few investigative teams have studied the relationship between snoring and GDM [4, 9–14]. A prospective cohort study of 189 healthy pregnant women reported frequent snoring (snoring ≥ 3 nights per week) was associated with an increased risk of GDM (OR = 6.7; 95% CI 1.4–33.8) [4]. Using a large cohort of 1, 673 US pregnant women, O’Brien et al administered a 1-hour oral glucose tolerance test using a 50-g load and reported that blood glucose levels at the 24- to 26-week gestation were higher in snorers compared to non-snorers (124.0 vs. 117.2 mg/dL, P < 0.001), as was the proportion of women with abnormal glucose levels, defined as ≥140 mg/dL (30.2% vs. 22.1%, P = 0.003) [11]. Additionally, O’Brien reported that habitual snoring was associated with a 1.67-fold increased risk of GDM (unadjusted OR = 1.67, 95% CI 1.10–2.52) [11]. Similar findings have been reported by Ge et al after examining 3, 079 Chinese pregnant women (adjusted RR = 1.66, 95% CI 1.09–2.53 for habitual snorer) after adjusting for covariates including pre-pregnancy BMI [14]. Conversely, Louis et al did not find an association between severe snoring and incident GDM; however, one must note that their study population was restricted to obese women [10]. Sharma et al, in their study of 209 Indian pregnant women, did not observe statistically significant association between snoring in early pregnancy and incident GDM [12]. In an earlier study, we reported no statistically significant association between habitual snoring and subsequent GDM risk (RR = 1.86; 95% CI 0.88–3.94) [9] which might be due to the small sample size and current study with s large sample size have found a statistically significant association.

Our observations of positive associations of snoring during early with risks of IGT and GDM, as well as findings reported by other investigators [4, 11, 13–14] who studied pregnant women, are consistent with reports from studies conducted among men and non-pregnant women [1, 6–7, 18]. For example, in analysis that included 69,852 US female nurses aged 40–65 years without diagnosed diabetes, cardiovascular disease, or cancer at baseline in 1986 after 10 years of follow-up, Al-Delaimy et al found that snoring was associated with risk of type II diabetes (for occasional snoring vs. non-snoring, RR = 1.48, 95% CI: 1.29–1.70; for regular snoring vs. non-snoring, RR = 2.25, 95% CI 1.91–2.66; p for trend < 0.001) after adjusting for age and body mass index [1]. Similar results were also found in a study that analyzed data from the National Health and Nutrition Examination Survey (NHANES) [6]. Snoring was associated with 10-year incidence of self-reported diabetes in 2,668 men aged 30–69 years in Sweden [18]. Further, joint exposure to both snoring and obesity had a 5-fold increased odds of diabetes compared with their absence [18]. Studies among men and women have also linked snoring and other sleep disturbances to increased risk of impaired glucose tolerance [19–21]. On balance, results from our study, and those of several others [4, 11, 14], indicate that self-reported snoring during early pregnancy is associated with an increased risk of IGT and GDM. These observations are consistent with findings from studies conducted among men and non-pregnant women as those who report habitual snoring have increased risks of developing type 2 diabetes [1, 6–7, 18].

Observed associations of early pregnancy snoring with incident IGT and GDM is biologically plausible. Available evidence suggests that individuals who snore tend to experience hypoxic stress and subsequent shallow and fragmented sleep time [4]. Snoring with resultant intermittent hypoxia facilitates the generation of reactive oxidative status contributing to increased oxidative stress, activation of a pro-inflammatory cascade, dyslipidemia, and insulin resistance [22]. Another plausible mechanism includes activation of the sympathetic nervous system leading to elevated catecholamine concentrations [23]; alterations in cortisol synthesis and release leading to alternations in sleep and glucose metabolism [24].

Our study has several important strengths including the fact that maternal early pregnancy snoring status was ascertained prior to screening and diagnostic testing of IGT and GDM, hence reporting bias is unlikely. However, several limitations merit consideration when interpreting our findings. Maternal snoring was obtained from self-report, and is susceptible to misclassification. Nonetheless, the use of self-reported snoring in epidemiological studies to detect sleep disordered breathing is well established [4, 6, 9, 11], maternal early pregnancy snoring status is susceptible to misclassification. Investigators have reported that self-reported snoring correlates well with objective measures from nocturnal polysomnography, especially in frequent snorers [25]. Self-reported infrequent or non-habitual snoring has not been shown to be a useful screen for sleep disordered breathing in large epidemiologic studies [26–27]. However, in our present study, we observed that infrequent snoring was associated with increased odds of GDM. One limitation of note in our study relates to the fact that were not able to separate the effect of habitual snoring before pregnancy from the effect of habitual snoring that emerged during early pregnancy. Additionally, the generalizability of our study results may be limited, as our cohort was primarily comprised of Non-Hispanic White and well-educated women. However, this concern is mitigated, in part, by the fact that our findings are similar with reports from studies conducted among pregnant women in China [14] and more racially and ethnically diverse populations [6].

## Conclusions

Consistent with studies of habitual snoring and GDM risk among pregnancy women [4, 11, 13–15], as well as studies of habitual snoring and type 2 diabetes among men and non-pregnant women [1, 6–7, 18], our study documented associations of habitual snoring during early pregnancy with increased odds of GDM; and that the odds were particularly elevated among overweight women. These findings, if confirmed, may have important implications for tailoring prenatal care for overweight pregnant women, and / or those with evidence of snoring, for preventing GDM. Further studies are needed to develop a more comprehensive understanding of the effects of sleep breathing disorders on maternal and newborn health outcomes.
